# Neurocysticercosis in Children with Seizures: A Cross-Sectional Study

**DOI:** 10.1155/2018/1030878

**Published:** 2018-05-21

**Authors:** Murli Manohar Gupta, Nagendra Chaudhary, Santosh Pathak, Nikhil Agrawal, Jaydev Yadav, Sandeep Shrestha, Om Prakash Kurmi, Baldev Bhatia, Kailash Nath Agarwal

**Affiliations:** ^1^Department of Pediatrics, Universal College of Medical Sciences, Bhairahawa 32900, Nepal; ^2^Centre for Population Health and Research (CPR), Bhairahawa 32900, Nepal; ^3^Chitwan Medical College, Bharatpur 44200, Nepal

## Abstract

**Background:**

Neurocysticercosis (NCC), a common cause of seizures in children from low and middle income countries (LMICs), if not diagnosed and treated early enough may lead to considerable morbidity and mortality. There is a lack of data on the prevalence of NCC and its clinical characteristics among those with seizure in South-Western Nepal.

**Aims and Objectives:**

To study the prevalence and clinical characteristics of NCC in children with seizures.

**Material and Methods:**

All children admitted to Universal College of Medical Sciences, a tertiary hospital in South-Western Nepal with seizures during 2014–16, were tested for NCC. NCC was diagnosed by neuroimaging [computerized tomography (CT) scan or magnetic resonance imaging (MRI)]. We used logistic regression to test the association between NCC with participants' characteristics and clinical symptoms.

**Results:**

Among 4962 in-patient children, 168 (104 boys and 64 girls) had seizures (138 with generalized tonic clonic seizures (GTCS) and 30 with focal seizures). 43% of children with seizures had CT scan confirmed NCC. The prevalence of NCC in the oldest children (13–16 years) was significantly greater (57.1% versus 15.6%) compared to the youngest (0–4 years) one (*p* < 0.001). Among 72 children with NCC, the proportions of children with vesicular, calcified, and colloidal stages were 76% (*n* = 35), 18% (*n* = 13), and 6% (*n* = 2), respectively. Children with focal seizures had 13% more NCC compared to those with GTCS but the result was statistically not significant. The adjusted odds of having NCC among 5–8 years, 9–12 years, and 13–16 years children were 6.6 (1.78–24.60), 11.06 (2.74–44.60), and 14.47 (3.13–66.96), respectively, compared to 0–4-year-old children. Reoccurrence of seizures within the first 3 months of taking antiepileptic drug in those with NCC was approximately 3 times higher compared to those without NCC (11% versus 4%, *p* = 0.084).

**Conclusions:**

This study shows that NCC contributes significantly to higher prevalence of seizures in children in South-Western region of Nepal.

## 1. Introduction

Neurocysticercosis (NCC), one of the most common helminthic infestations of the brain, is highly prevalent in low and middle income countries (LMICs). It is one of the most common causes of acquired epilepsy and neurological morbidity in children [1].

A community based survey conducted in 300 patients from Morang district of Nepal reported that NCC was the main risk factors in about 7.3% prevalent seizure cases as was in another study from Western Nepal that also reported NCC as the common neuroimaging findings in abnormal brain scans [2, 3].

Central nervous system (CNS) involvement is seen in 60–90% of infested patients with cerebrum and cerebellum as common sites but cysticerci may involve brainstem, basal ganglion, thalamus, and lateral sinus as well [4].

NCC is common in the communities with poor hygiene and sanitation where untreated human faeces are used in the field/garden as fertilizer. Vegetarians might get infected by eating raw vegetables contaminated with ova or proglottids of* Taenia solium* whereas nonvegetarians may get infected after eating improperly cooked pork meat [5]. This study was thus conducted to study the prevalence, the clinical profile, and the radiological findings of NCC in children presenting with seizures.

## 2. Material and Methods

This study recruited children aged 0–16 years from 1 August 2014 to 31 July 2016 at a tertiary hospital of South-Western Nepal. All cases with seizures admitted to pediatric ward and pediatric intensive care unit were enrolled following written and verbal consent. Seizure was defined as a transient occurrence of signs and/or symptoms resulting from abnormal excessive or synchronous neuronal activity in the brain (as per International league against epilepsy (ILAE) classification). Neurocysticercosis was diagnosed by the revised criteria as described by Del Brutto [6]. Radiological classification of NCC was done as per Escobar's pathological staging system [7]. Children not presenting with seizures and/or encephalopathy or children with seizures and/or encephalopathy where neuroimaging (computed tomography or magnetic resonance imaging) could not be carried out were excluded from the study. To rule out tubercular infection of brain, a complete blood count, Mantoux test, erythrocyte sedimentation rate (ESR), X-ray chest, and gastric aspirate for acid fast bacilli (AFB) were done in all patients having NCC in CT or MRI scan. The study was approved by the Universal Medical Colleges institute's ethical review board (IRB).

Sample size (*n* = 162) was calculated “anticipating 11.9% prevalence of neurocysticercosis in epilepsy patients [8] to fall within 10% points of true proportion with 95% confidence interval.” Data was recorded in predesigned pro forma. Patients were followed up in 3 months' time following administration of antiepileptic drugs (AEDs). Those patients who failed to attend OPD clinic in three months of initial diagnosis and treatment were contacted by telephone and enquired regarding the breakthrough seizures at 3 months.

All data was entered in Microsoft excel chart. Data was analyzed using Stata v13. Descriptive analysis was done as percentage and mean. For categorical variables, *p* value for heterogeneity and, for more than two variables, *p* value for trend were calculated using chi-square test. Logistic regression was done to calculate the odds of having NCC in children presenting with a particular clinical symptom after adjusting for various sociodemographic factors and other symptoms.

## 3. Results

**Figure 1:**
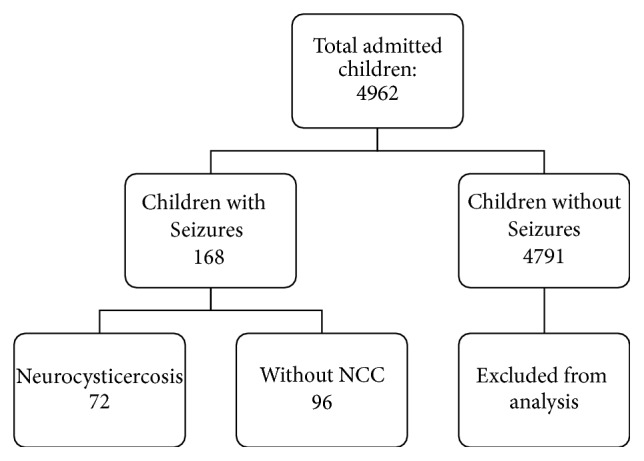
Flowchart showing work plan of admitted children.

Out of 4,962 children admitted to either pediatric ward or pediatric intensive care unit during the study period, 168 (3.4%) had seizures. 43% (*n* = 72) of the total children (age: 2 months to 16 years) with seizures had CT scan confirmed NCC (Figure 1). Mean age of children having NCC was 9.8 ± 3.9 years and with male-female ratio of 1.4 : 1. The prevalence of NCC increased significantly in older children (13–16 years) compared to youngest children (0–4 years) from 15.6% to 57.1% (*p* < 0.001) (Tables 1 and 3). Among 72 children with CT scan confirmed NCC, 76% (*n* = 55) had vesicular, 18% (*n* = 13) calcified, and the remaining 6% colloidal (*n* = 2) or nodular (*n* = 2) subtypes (Table 2).

Children with focal seizures had 12.8% more NCC compared to those with GTCS but the result was statistically not significant (*p* = 0.201). Unconsciousness (*n* = 38, 40%), vomiting (*n* = 24, 25%), fever (*n* = 17, 18%), and headache (*n* = 13, 14%) were four leading clinical manifestations in children with NCC. Similarly, among many clinical symptoms, the odds of not having NCC in those with fever were 3-fold higher compared to non-NCC children (*p* = 0.025). The adjusted odds of having NCC among 5–8-year-old, 9–12-year-old, and 13–16-year-old children compared to 0–4-year-old children were 6.6 (1.78–24.60), 11.06 (2.74–44.60), and 14.47 (3.13–66.96), respectively (Table 3).

Phenytoin was the drug of first choice (58%) among those with or without NCC followed by sodium valproate as the second choice (32.7%). The result also shows that the reoccurrence of seizures within the first 3 months of taking antiepileptic drug in those with NCC is approximately 3 times higher compared to those without NCC (11% versus 4%, *p* = 0.084) (Table 1).

## 4. Discussion

Neurocysticercosis is an important cause for hospital admissions in children with seizures in Nepal. The prevalence of seizures in children in the present study was 33.8 per 1000 children and 43% (*n* = 72) of children with seizures had NCC.

A similar study carried out in Kathmandu, a hilly region of Nepal, reported that 16% of children with seizures had NCC and various studies from India have reported its prevalence to be in the range of 12–35% suggesting wide variability of NCC in different geographical regions [8–11]. The high prevalence may be due to number of factors such as high consumption of pork meat, rearing of pigs, consumption of raw vegetables, and lack of personal and environmental hygiene and sanitation. Lack of proper meat inspection and improper husbandry practice may also be an important factor for its high prevalence in LMICs like Nepal. Although the main objective of our study was to find the prevalence of NCC in patients with seizures, interestingly, we did not encounter NCC patients without seizures during the study period as was also the case in other study carried out by Shrestha et al. [12] in Nepal and another study from the same region [13] reported that 91% of children with NCC had seizures where 85% of seizure semiology was focal in nature.

The proportion of girls with NCC in our study was insignificantly higher compared to boys as was also reported in other studies [14, 15] but the result is inconsistent as many studies [16–20] have reported higher proportion of NCC among boys with seizures. The reason for increased occurrence of NCC in females could be because of their traditional roles in domestic work, including in agriculture, rearing pet animals, and poor hand hygiene with poor sanitation.

We noticed increased prevalence (59.7%) of NCC amongst the older children (9–16 years group) compared to only 9.7% in younger (0–4 years) age group. Our results are consistent with findings from some previous studies [8, 21]. A study carried out by Shrestha et al. [12] from Nepal in school going children reported the highest prevalence of NCC (48%) in the 10–15 years age groups. The findings of higher prevalence has been consistent among many other studies conducted in different part of Nepal [13, 17]. The prevalence increased after 7 years in their study and no NCC in children under 2 years. The reason of lower prevalence of NCC in younger age groups could be due to prolonged incubation period of* Taenia solium *or could probably be due to their dietary habits as they are less likely to be exposed to outside foods in comparison to adolescents and teens.

NCC may be asymptomatic but may produce a broad range of clinical manifestations. Seizure is by far the most common clinical manifestation. Less common manifestations include headache alone; symptoms of raised intracranial pressure (ICP); altered mental status (cysticercal encephalitis); and acute psychosis. Only a minority of patients present with cranial nerve palsies or other focal neurological deficits. The clinical spectrum of the disease depends upon the location, number, and viability of the cysts as well as host response.

In the present study, GTCS occurred in around three-fourths of NCC cases, while the remaining one-fourth had focal seizures. Our findings were in line with some earlier findings [22, 23]. The type of seizure semiology was different in different studies from various countries including Nepal. Some studies supported the occurrence of focal seizures, while others suggested the occurrence of generalized seizures as in the present study. This suggests that NCC can present with seizure of any semiology.

Loss of consciousness, vomiting, fever, and headache were the top four leading clinical manifestations in children having neurocysticercosis with seizures in our present study. A study by Gauchan et al. [17] from Western Nepal too had similar clinical manifestations as was found in our study and both the studies found that loss of consciousness and headache were the leading clinical manifestations. Similar clinical manifestations are reported by other studies [13, 16, 24].

The most common stage of neurocysticercosis observed in our study was vesicular stage. Majority of the vesicular stage NCC had GTCS but the distribution in the semiology of seizures with respect to the stages of NCC was statistically insignificant (*p* = 0.538). In a study carried out in Western Nepal, the occurrence of active stages among NCC cases was 73% as was in our study and some other studies [17, 22]. In a study conducted by Shrestha et al. [25], it was found that the lesions found in CT scan were mostly in a transitional stage (61.22%), whereas perilesional edema and scolex within the lesion were noted in 67% and 18% of the cases, respectively. Talukdar et al. [26] reported similar CT findings. This localized brain edema circumscribing ring enhancing/calcified lesions of NCC can be considered to be the cause of seizures, which may be caused by an inflammatory response due to the liberation of antigens by the calcified cyst itself during its remodeling process.

MRI is more sensitive in identifying the scolex within the cyst and in detection of intraventricular and subarachnoid lesion, whereas CT head is more sensitive in identifying calcified granuloma. MRI head was done in 13 patients; one patient had NCC (vesicular stage), whereas 4 patients had normal findings and 8 had other findings. We could not get MRI done in all 168 patients due to lack of financial resources. Therefore, we could not compare the sensitivity and specificity of both diagnostic tools (CT versus MRI) for detecting NCC. Since CT head is easily accessible and its cost is comparatively cheaper in comparison to MRI of the brain, it has been proven to be preferred diagnostic tool for diagnosing NCC in resource limited countries.

Out of 72 NCC patients, 64 patients were seizure free, whereas eight patients had recurrence of seizures at the end of 3-month follow-up period. 11% of total NCC cases had breakthrough seizures. The seizure recurrence in patients treated with albendazole in a study done by Gogia et al. [27] was 8–13%, whereas it was high (56%) in another study carried out by Garcia et al. [28].

## 5. Conclusion

In summary, our study supports the earlier findings that neurocysticercosis usually affects children with increasing prevalence in teenagers and adolescents. The proportion of females with NCC was higher than males. The most common clinical manifestation of NCC is seizure where GTCS is common compared to focal seizures. Loss of consciousness and headache were other common clinical findings in NCC. The occurrence of fever was significantly low in cases of NCC. Vesicular stage of NCC followed by calcified stage was the common radiological findings in CT scan head.

Any child presenting with a first episode of afebrile seizure should be evaluated with an imaging of brain (CT scan/MRI), especially if they present after 3 years of age, provided that other causes of seizure are ruled out.

## Figures and Tables

**Table 1:** Sociodemographic characteristics, neuroimaging, and antiepileptic drug use in children with NCC.

Characteristics	Number	NCC *n* (%)		*p* value
		Yes	No	
*Number of patients N (%)*	168	72 (42.9)	96 (57.1)	

*Gender*				
Male	104	42 (40.4)	62 (59.6)	0.409
Female	64	30 (46.9)	34 (53.1)	

*Age (in years)*				
0–4	45	7 (15.6)	38 (84.4)	<0.001
5–8	44	22 (50.0)	22 (50.0)	
9–12	44	23 (52.3)	21 (47.7)	
13–16	35	20 (57.1)	15 (42.9)	

Mean (±SD)	168	9.8 (3.9)	7.0 (4.6)	

*Religion*				
Hindu	154	66 (42.9)	88 (57.1)	1.000
Muslim	14	6 (42.9)	8 (57.1)	

*CT scan results*				

Normal	73	0 (0.0)	73 (100.0)	0.402
NCC	72	72 (100.0)	0 (0.0)	
Others	23	0 (0.0)	23 (100.0)	

*MRI scan results*				

Normal	4	1 (25.0)	3 (75.0)	0.296
NCC	1	1 (100.0)	0 (0.0)	
Others	8	0 (0.0)	8 (100.0)	

*Antiepileptic drugs*				

Phenytoin (P)	98	42 (42.9)	56 (57.1)	
Sodium valproate (V)	55	26 (47.3)	29 (52.7)	
Both P + V combined	11	4 (36.4)	7 (63.6)	
More than two drugs	4	0(0.0)	4 (100.0)	

*Breakthrough seizures (3 months)*	12	8 (66.7)	4 (33.3)	0.084

**Table 2:** Different stages of NCC in children in relation to types of seizures.

Neurocysticercosis	Number	GTCS *n* (%)	Focal seizure *n* (%)	*p* value
No	96	82 (85.4)	14 (14.6)	
Yes	72	56 (77.8)	16 (22.2)	0.201

*Stages in CT scan*				
Vesicular	55	44 (80.0)	11 (20.0)	
Calcified	13	9 (69.2)	4 (30.8)	0.538
Colloidal	2	1 (50.0)	1 (50.0)	
Nodular	2	2 (100.0)	0 (0.0)	

**Table 3:** Relationship between NCC with types of seizures, various sociodemographic characteristics, and clinical symptoms.

Characteristics	*N*	Odds ratio (95% CI)	*p* value
*Seizure types*			
GTCS	138	Reference	
Focal	30	1.37 (0.46–4.10)	0.573
*Gender*			
Male	104	Reference	
Female	64	1.81 (0.69–4.76)	0.229
*Religion*			
Hindu	154	Reference	
Muslim	14	1.57 (0.32–7.75)	0.579
*Age groups (in years)*			
0–4	45	Reference	
5–8	44	6.60 (1.78–24.43)	**0.005**
9–12	44	11.06 (2.74–44.60)	**0.001**
13–16	35	14.47 (3.13–66.96)	**0.001**
*Clinical symptoms*			
Fever	67	0.34 (0.13–0.87)	**0.025**
Meningeal irritation	7	0.19 (0.02–2.36)	0.198
Headache	27	0.63 (0.21–1.91)	0.416
Unconsciousness	93	0.84 (0.35–2.00)	0.695

Odds ratio was calculated using multivariate logistic regression. The comparator groups for various clinical symptoms are those without symptoms. For age group subclassification, the reference age groups were 0–4 years.

## References

[1] Rajshekhar V. (2016). Neurocysticercosis: Diagnostic problems & current therapeutic strategies. *Indian Journal of Medical Research*.

[2] Rajbhandari K. C. (2004). Epilepsy in Nepal. *Canadian Journal of Neurological Sciences*.

[3] Adhikari S., Sathian B., Koirala D. P., Rao K. S. (2013). Profile of children admitted with seizures in a tertiary care hospital of Western Nepal. *BMC Pediatrics*.

[4] Handique S. K., Das R. R., Saharia B., Das P., Buragohain R., Saikia P. (2008). Coinfection of Japanese encephalitis with neurocysticercosis: An imaging study. *American Journal of Neuroradiology*.

[5] Singhi P., Gahlot A. Pediatric neurocysticercosis: current challenges and future prospects. *Pediatric Health, Medicine and Therapeutics*.

[6] Del Brutto O. H. (2012). Diagnostic criteria for neurocysticercosis, revisited. *Pathogens and Global Health*.

[7] Escobar A., Palacios E., Rodriguez-Carbajal J., Taveras J. M. (1983). The pathology of neurocysticercosis. *Cysticercosis of the central nervous system. Charles C. Thomas*.

[8] Mondal M., Biswas B., Roy A. (2015). A retrospective analysis of variability of clinical presentations and brain imaging findings in children with neurocysticercosis in rural population of West Bengal. *Asian Journal of Medical Sciences*.

[9] Ojha R., Shah D., Shrestha A. (2015). Neurocysticercosis in Nepal: a retrospective clinical analysis. *Neuroimmunology and Neuroinflammation*.

[10] Goel D., Dhanai J. S., Agarwal A., Mehlotra V., Saxena V. (2011). Neurocysticercosis and its impact on crude prevalence rate of epilepsy in an Indian community. *Neurology India*.

[11] Kumar A., Khan S. A., Khan S., Das S., Anurag, Negi K. S. (2006). A study of neurocysticercosis in the foothills of the Himalayas. *International Journal of Infectious Diseases*.

[12] Shrestha S., Dhungana S., Shrestha A. (2013). Neurocysticercosis in Children at GMC, Charak Hospital, Pokhara. *Journal of Chitwan Medical College*.

[13] Shrestha B. M. (2008). Childhood neurocysticerosis: Clinico-radiological profile and outcome. *Journal of Nepal Paediatric Society*.

[14] Ruiz-García M., González-Astiazarán A., Rueda-Franco F. (1997). Neurocysticercosis in children. *Child's Nervous System*.

[15] Ferreira L. S., Zanardi V. A., Scotoni A. E., Li M., Guerreiro M. M. (2001). Childhood epilepsy due to neurocysticercosis: A comparative study. *Epilepsia*.

[16] Singhi P., Ray M., Singhi S., Khandelwal N. (2000). Clinical spectrum of 500 children with neurocysticercosis and response to albendazole therapy. *Journal of Child Neurology*.

[17] Gauchan E., Malla T., Basnet S., Rao K. S. (2011). Variability of presentations and CT-scan findings in children with neurocysticercosis. *Kathmandu University Medical Journal*.

[18] Joshi D. D., Bista P. R., Ito A., Yamasaki H. (2007). Present situation of porcine taeniasis and human cysticercosis in Nepal. *Southeast Asian journal of tropical medicine and public health*.

[19] Morales N. M. O., Agapejev S., Morales R. R., Padula N. A. M. R., Lima M. M. F. (2000). Clinical aspects of neurocysticercosis in children. *Pediatric Neurology*.

[20] Singhi P. D., Dinakaran J., khandelwal N., singhi S. C. (2003). one vs two years of antiepeliptic therepy in children with single small enchancing CT lesion. *Journal of Tropical Pediatrics*.

[21] Thakur L. C., Anand K. S. (1991). Childhood neurocysticerosis in South India. *The Indian Journal of Pediatrics*.

[22] Basu S., Ramchandran U., Thapliyal A. (2007). Clinical profile and outcome of pediatric neurocysticercosis: A study from Western Nepal. *Journal of Pediatric Neurology*.

[23] Nash T. (2012). Edema surrounding calcified intracranial cysticerci: Clinical manifestations, natural history, and treatment. *Pathogens and Global Health*.

[24] Kalra V., Sethi A. (1992). Childhood Neurocysticercosis-Epidemiology, Diagnosis and Course. *Pediatrics International*.

[25] Shrestha B., Mainali P., Sayami S., Shrestha O. K. (2013). Clinico-radiological aspects of neurocysticercosis in pediatric population in a tertiary hospital. *Journal of Nepal Medical Association*.

[26] Talukdar B., Saxena A., Popli V. K., Choudhury V. (2002). Neurocysticercosis in children: Clinical characteristics and outcome. *Annals of Tropical Paediatrics*.

[27] Gogia S., Talukdar B., Choudhury V., Singh Arora B. (2003). Neurocysticercosis in children: Clinical findings and response to albendazole therapy in a randomized, double-blind, placebo-controlled trial in newly diagnosed cases. *Transactions of the Royal Society of Tropical Medicine and Hygiene*.

[28] Garcia H. H., Pretell E. J., Gilman R. H. (2004). A Trial of Antiparasitic Treatment to Reduce the Rate of Seizures Due to Cerebral Cysticercosis. *The New England Journal of Medicine*.

